# Odontological Society of Pennsylvania

**Published:** 1894-04

**Authors:** 


### ODONTOLOGICAL SOCIETY OF PENNSYLVANIA.

At a meeting of the Society, held at 1228 Walnut Street, the President, Dr. E. T. Darby, in the chair.

After the transaction of routine business, Dr. S. G. Perry, of New York, read a paper upon the proper care of the teeth to the fifteenth year.

(For Dr. Perry's paper, see page 148.)

### DISCUSSION.

*Dr. Jarvie*, New York.—This is a case in which the last shall be first, for I was the last comer, and, unfortunately, I was not in the room when Dr. Perry commenced his paper, and so did not hear all of it; but it is a most interesting question, and has been ably treated. It is a subject that does not receive the consideration it deserves. I think we should so treat the teeth of children that at middle age they will have a good, wholesome set of teeth, and to accomplish such a result requires good judgment. The paper stated that observing and directing the development of a set of teeth was, perhaps, one of the most interesting things that occurs in the dentist's professional career. That has been my experience, and, in deciding upon the management of a child's teeth in any given case, I have always had in view the condition I hoped to have them in when the patient should attain middle age.

To accomplish a desirable result, we are sometimes embarrassed by the parents, whom it is desirable to be made to understand why we use materials to fill the teeth of their children which are necessarily temporary in their character, and which must be renewed from time to time, rather than to use gold, which many have an idea is the best filling-material for all teeth, and which, once inserted, ought to last forever. All teeth of young children, and those of delicate organization not young, ought almost invariably to be filled with the plastics or with tin, and such fillings must frequently be added to or replaced as circumstances demand. Gutta-percha, oxyphosphate of zinc, and tin are by far the best substances to use, yet I should be very sorry to be without one filling-material that has been severely denounced by the essayist, and that is copper amalgam. I have had one or two experiences such as Dr. Perry related, and have seen many others; but I still think copper amalgam is very valuable as one of our filling-materials.

There are certain classes of children's teeth—I mean quite young children, in which we can save the temporary molars for a few years by the use of that material—that we cannot save in any other way. I have one or two cases in mind,—a child three and a half years of age was brought to me with the worst teeth I have ever seen in a child,—one of the most healthy and vigorous-looking, yet at this age not a sound tooth. The superior incisors, all four of them, were broken off. The gums were healthy, but the teeth had become exceedingly sensitive, so much so that the child masticated food with difficulty, and the condition was becoming serious. I removed the decay in the molars as well as I could, yet imperfectly, keeping the cavities as dry as possible without the rubber dam, as I could not apply that, and saturated the surfaces with carbolic acid for a few minutes. Then I filled with copper amalgam, and it seemed to have apparently some adhesive property, for it remained where I had hardly dared expect it to.

Another case was somewhat similar,—the child five or six years of age and the temporary molars in the same condition. This last case was treated about two years ago, and I see the child frequently, and the fillings remain good, very much better than I had any idea they would when I tried it, for I used it largely as an experiment. The child, from masticating with difficulty and pain, found she could masticate with comfort, and the relief seemed marvellous to her. I am the best friend she has, and she comes to me with delight. I do not think I could have accomplished the same result with any other material than copper amalgam. I use it frequently in temporary teeth, and it serves a purpose accomplished by no other material.

I want to emphasize all that the essayist said in regard to the use of tin-foil in the fissures of the temporary molars, but more particularly, perhaps, in the sixth-year molars. I think it is by all odds the most valuable material we have for such cases. The fissures can be readily removed, where the decay has not progressed to any great extent, and easily filled with tin-foil. I use No. 3. It works almost as plastic and is almost as readily applied as amalgam. I had the pleasure of seeing some block tin prepared by your president the other night which had a quality I had never seen before. It was soft, yet cohesive, and was in the form of shavings, and will undoubtedly prove of value.

*Dr. Hall*, of China.—I am fully conscious of the fact that you will attach more importance to the unique place of my residence than you will to anything I might say to you upon the subject of the paper that has been read.

Though the paper stopped at the fifteenth year and assumed the care of the teeth to that time, it seems to me that Dr. Perry has touched about every point that constitutes the perfect salvation of the temporary and permanent teeth, for, having carried the patient up to the fifteenth year with such treatment as he describes, the permanent teeth are perfectly safe. The discussion of the material that enters into the care of the teeth of young or old is, I believe, of minor importance compared with the true welfare of the patient. The wish to keep the teeth as Nature meant they should be kept is evidenced in every line of his paper. That which the artist used in mixing his colors applies most fitly to everything that the dentist does in the care of the teeth. And, whilst I am distinctly in favor of certain facts of detail and the wisdom of deciding upon certain things as facts from which we may not depart at all except in the line of experimentation, I am still willing to believe that great laxity is permissible in the choice of materials, from copper amalgam to gutta-percha, or anything else. We all know that Dr. Perry is not in the habit of presenting in his practice or before societies anything that is not of the very best. I feel very much pleased that I have been here, and I am more than flattered to think that, after an absence of more than fifteen years from the profession, I can truthfully say that I have, in a large measure, had the pleasure of agreeing with Dr. Perry in methods and most of the points that he has brought out to-night, and it gives me a great deal of satisfaction to find myself fairly in line with those who are older and banded together in this country for that which is the very best in the preservation of the teeth.

Dr. Woodward expressed himself as much pleased with the paper, having come from New York to listen to Dr. Perry's views on this important topic.

*Dr. Truman.*—Dr. Perry's paper is entirely in accord with my own views in regard to this subject, and are those I have held for the last twenty or twenty-five years. Years ago, when discussing the question of the treatment of children's teeth, I felt myself practically alone when advancing the same ideas that Dr. Perry has formulated to you so thoroughly to-night.

I, however, go further than he does in this matter, for I am almost prepared to assert that it is malpractice for any individual to place gold fillings in children's teeth from eleven to sixteen years of age. I know that is a strong statement, and perhaps I should qualify it to some extent by the assertion that some teeth will bear it; but we cannot reason upon exceptional teeth. The teeth that are sufficiently dense at that age to bear such fillings are extremely few.

What is the philosophy of this? You all comprehend the fact that a tooth at this age of life is not fully developed. That all the tubuli, histologically speaking, are very much larger than they will be at a later period; and if you place a good conductor in the teeth, as gold, irritation will be produced, and the irritation upon the peripheral ends of the tubes will be carried to the pulp, producing inflammation and final destruction. If this be the case (and I cannot see that it is possible for any one to deny it, knowing the minute anatomy of the tooth-structure), are we justified in ever using gold in that class of teeth? I have, therefore, declined to use gold up to the twentieth year, except in rare cases, notwithstanding the frequent objection of parents. If parents are permitted to dictate to us, we have mistaken our calling. I have had some such difficulty, but I always say, You must accept my word in the matter or it would be better to take the child elsewhere. This I regard as the only course to pursue in these cases until greater density is secured. The expression that "whatever tooth was worth filling was worth filling with gold" has lost its force, and we are coming down to a philosophical method of treating teeth,—not alone those of children, but all teeth.

Dr. Perry seems inclined to fill the canals of deciduous teeth occasionally. I should say, never fill them. I do not see the necessity for this operation. If a proper antiseptic be used, it will be all that is required. [Dr. Perry here stated that Dr. Truman had misunderstood him, that he did not state that he filled temporary tooth roots.]

Dr. Truman explained that he so understood it and was gratified to know he was in error.

*Dr. Faught.*—But one idea has entered my mind while listening to this discussion to which I would like to give expression. We have heard the old story often repeated. Men of unquestioned integrity and observation tell us one thing, and men of equal integrity and observation and learning tell us another thing. The question ever is, What is accepted practice? We find out, after all, that the latitude as to how to care for the teeth to the fifteenth year is not a fixed quantity. That it is not malpractice to do certain things we have had very plainly exemplified here to-night; but it is very desirable to arrive at definite methods of treating teeth. Dentistry can never become the fixed thing it should be in the eyes of the public until that time. Here is full reason why it is so difficult to get our patients to understand that a filling once put in a tooth will not preserve that tooth forever, and the reason why we find it so hard to make parents understand why we use plastic fillings instead of gold up to a certain age. Our patients go out from us and rub against each other and find that the majority of men in dentistry are not a unit in the matter, and they get down to the conclusion that gold must be used in all cases, and they come to us with a determined effort that will make it difficult to care for these children at this age.

*Dr. Jefferis.*—Dr. Perry started out by giving us what we like to hear,—his own views, and not a dogmatic expression of them. If I have any difference with him it is in the use of amalgam. I use amalgam with a small percentage of copper in it,—not the copper amalgam as ordinarily understood. Also in the use of pink gutta-percha on the approximal surfaces of the temporary molars where they are decayed. In making a filling between these teeth I frequently use it, and find that it answers the purpose not only in arresting the decay, but in protecting the gum from the impaction of food between the teeth, which separate hard fillings will not do. The teeth naturally fall apart as the jaw grows to accommodate the larger second teeth, and if the space is filled between them when decayed with the pink gutta-percha, it will protect the gums while arresting decay of the teeth. And further, when the molars are devitalized, while I extract the pulp as well as may be, I place a disk over the pulp cavity and fill the cavity in crown only, in every case drilling a vent at the cervical buccal margins.

*Dr. Boice.*—I use gutta-percha and tin, and very little oxyphosphate; but my principal difficulty is in getting good gutta-percha. I once made a statement at the Pennsylvania State Society that I would give a hundred dollars for an ounce of good gutta-percha. I believe I would now give a hundred dollars for an ounce of the same material.

*Dr. Bonwill.*—It is with pleasure I greet such practical men as the essayist, Dr. Perry. His suggestions are from honest convictions of sight. He is an enthusiast in his profession and desires our consideration, and his teachings are generally good,—very good. He is a safe operator to follow, and while we differ on some matters, yet I can say in sincerity, follow him. Think for yourself at the same time, let be said what may and from whomsoever it may come.

*Dr. McQuillen.*—Dr. Perry mentioned the difficulty in satisfying parents. I think we should follow our own conscience in what we do and not allow them to enter into a discussion. When I say I am going to fill a tooth with a certain thing there is no discussion. It goes in.

*Dr. Bassett.*—It seems to me the paper is very exhaustive. I have followed Dr. Perry's ideas closely in my own practice. In regard to the treatment of teeth by the use of plastics before the fifteenth year, I may go further and say with Dr. Truman that I consider the use of plastics, of the kind mentioned, necessary until the teeth are thoroughly solidified, in spite of the protests of parents who consider that a tooth once filled should be always filled, and that gold is the only filling to use. I must continue to use gutta-percha where I think it is of the best to preserve the teeth from decay which are below the average in quality, and withholding the use of gold and other hard materials until the teeth are thoroughly solidified.

*Dr. Roberts.*—I endorse Dr. Perry's statement. Copper amalgam I have no use for; it is too treacherous, and also leads to the destruction of the teeth even after the crowns are gone.

Dr. Peirce said he thought the paper was overflowing with good common sense.

*Dr. Milliken.*—The gentleman from Shanghai, Dr. Hall, said it was an excellent paper and covered all the points. I fully agree with him as far as the paper goes, but I do not think it has gone far enough. I think as a profession we are apt to confine ourselves to filling-materials, which I regard as a mistake. When patients come to us we should quietly take them in; not financially, but physically. We should make strict inquiries into their mode of life, especially if they be young, and as we go on from month to month, watch to see if there is any change in their health. I think if we made a practice of it we could recognize any changes in their condition which could be corrected by calling their own or their parents' attention to it. Very few of us ever think of such a thing as inquiring how many hours a day the patient spends in the school-house, and how many hours in the open air; what they eat and drink and how many hours they sleep; whether the sleep be sweet and restful or disturbed and restless. Such things do not seem to cross our minds. As an instance of that, I had a little patient who has been in the habit of coming about every three months (a pretty, healthy-looking child). She had never been afraid of me, and I had always managed to get along nicely with her. I noticed when she came two months ago that she was nervous and fidgety, and the moment I took up my mouth mirror she nervously asked me what I was going to do. The question surprised me. I inquired if she slept well, if her appetite was good, etc.; she replied, "Yes." Upon questioning, I found that she didn't go out to play in the air much, and I sent her away and told her to tell her mother to come to see me. The mother came the next day, and I asked her if she was aware that her child was running down. She was surprised and answered, "No!" She said the child did not sleep well, but was restless and dreamed a great deal, and that her appetite had not been good for several months. I suggested that she consult her physician, and advised that the child be taken from school and sent away or kept in the open air for a month or so. She took my advice, keeping the child away from school for about a month, and it was wonderful what a change took place, not only in her mouth, but also in her general health.

I think that the best way to preserve both the temporary and permanent teeth is to preserve the general health, a plan that has not been adopted, unfortunately, in our profession. It seems to be a subject never, or very seldom, given a thought.

The President introduced Dr. Crouse, who on behalf of the Dental Protective Association solicited the members to take shares of stock in order to raise one hundred thousand dollars, with which to equip a manufactory for making dental appliances. The shares being those of an incorporated association created under the laws of the State of Illinois.

Adjourned.

